# Divergent effects of listening demands and evaluative threat on listening effort in online and laboratory settings

**DOI:** 10.3389/fpsyg.2024.1171873

**Published:** 2024-01-25

**Authors:** Peter J. Carolan, Antje Heinrich, Kevin J. Munro, Rebecca E. Millman

**Affiliations:** ^1^School of Health Sciences, Manchester Centre for Audiology and Deafness, University of Manchester, Manchester, United Kingdom; ^2^Manchester University Hospitals NHS Foundation Trust, Manchester Academic Health Science Centre, Manchester, United Kingdom

**Keywords:** listening effort, motivation, speech perception, social pressure, remote testing

## Abstract

**Objective:**

Listening effort (LE) varies as a function of listening demands, motivation and resource availability, among other things. Motivation is posited to have a greater influence on listening effort under high, compared to low, listening demands.

**Methods:**

To test this prediction, we manipulated the listening demands of a speech recognition task using tone vocoders to create moderate and high listening demand conditions. We manipulated motivation using evaluative threat, i.e., informing participants that they must reach a particular “score” for their results to be usable. Resource availability was assessed by means of working memory span and included as a fixed effects predictor. Outcome measures were indices of LE, including reaction times (RTs), self-rated work and self-rated tiredness, in addition to task performance (correct response rates). Given the recent popularity of online studies, we also wanted to examine the effect of experimental context (online vs. laboratory) on the efficacy of manipulations of listening demands and motivation. We carried out two highly similar experiments with two groups of 37 young adults, a laboratory experiment and an online experiment. To make listening demands comparable between the two studies, vocoder settings had to differ. All results were analysed using linear mixed models.

**Results:**

Results showed that under laboratory conditions, listening demands affected all outcomes, with significantly lower correct response rates, slower RTs and greater self-rated work with higher listening demands. In the online study, listening demands only affected RTs. In addition, motivation affected self-rated work. Resource availability was only a significant predictor for RTs in the online study.

**Discussion:**

These results show that the influence of motivation and listening demands on LE depends on the type of outcome measures used and the experimental context. It may also depend on the exact vocoder settings. A controlled laboratory settings and/or particular vocoder settings may be necessary to observe all expected effects of listening demands and motivation.

## Introduction

Listening effort (LE) refers to the mental work required to understand speech in challenging situations, such as when struggling to hear a conversation in a busy café. Pichora-Fuller and colleagues define LE as “*the deliberate allocation of mental resources to overcome obstacles in goal pursuit when carrying out a [listening] task*” (Pichora-Fuller et al., 2016, p. 10S), a definition that emphasises the pivotal role of motivation in effortful listening. In the context of listening, motivation pertains to the social benefits of listening which drives individuals to continue to exert effort in difficult listening environments (Matthen, 2016; Hughes et al., 2018).

The interactive role of task demands, motivation and other factors, such as resource availability for LE was further clarified by Kruglanski and colleagues within the framework of Cognitive Energetics Theory (Kruglanski et al., 2012). They proposed two opposing forces, a driving and a restraining force that affect LE. The driving force comprises an individual’s motivation to achieve a task goal and their availability of resources to do so. The listening demands of the task, the individual’s tendency to conserve resources and alternative goals competing for resources combine to form a “restraining force” against exerting LE. Therefore motivation and task difficulty interact in determining LE, such that motivation is a more important factor when tasks are harder (though not impossible) compared to when tasks are easier.

Motivation can be operationalised in ways that allow for external manipulation (financial reward, evaluative threat, feedback, perceived competence) or in ways that use natural variations of individual traits which affect levels of motivation. Evaluative threat manipulations place participants under psychosocial stress due to the “threat” of an upcoming test (Carolan et al., 2022). Evaluative threat can be created by informing participants that they would be tested on stimuli presented to them (Picou and Ricketts, 2014), or that their performance would be compared to their peers (Carrillo-de-la-Pena and Cadaveira, 2000). Another option is to inform participants that their data will not be usable unless they reach a certain performance threshold. This operationalisation of evaluative threat may be a particularly useful in an online setting and for subjective measures of LE (Zekveld et al., 2019, see Carolan et al., 2022). In a meta-analysis investigating the influence of motivational factors on LE, evaluative threat manipulations of motivation yielded the largest effect size, Evaluative threat may increase LE investment due to participants seeking to avoid negative evaluation (Picou and Ricketts, 2014; Zekveld et al., 2019). Higher levels of evaluative threat have been shown to result in greater self-rated work (Picou and Ricketts, 2014; Zekveld et al., 2019), faster reaction times (RTs) (interpreted as greater arousal) (Carrillo-de-la-Pena and Cadaveira, 2000) and reduced self-rated tiredness (Picou and Ricketts, 2014).

In addition to motivation, resource availability is another driving factor for LE. It allows for the use of more effective cognitive strategies, increasing the likelihood of goal attainment (Kruglanski et al., 2012). Task performance may therefore increase with greater LE expenditure. The Ease of Language Understanding Model proposes a similar idea: individuals recruit working memory resources to understand speech in challenging situations (Rönnberg et al., 2019). Individuals with higher working memory capacity should have greater resource availability, a stronger driving force towards exerting LE and ultimately achieve a better level of performance. No previous studies have considered the effects of evaluative threat and resource availability on listening effort together.

Finally, besides LE, listening-related fatigue is an important concept to understand in the context of speech perception in challenging situations. Listening-related fatigue is defined as self-perceived “*extreme tiredness resulting from effortful listen*ing” (McGarrigle et al., 2014, p. 434). According to the Motivational Control Model, fatigue signals to individuals that they need to reduce effort on the current task and redirect resources towards alternative actions with greater utility (e.g., higher rewards or lower effort costs). Fatigue may be an adaptive state which ensures the efficient allocation of effort (Hockey, 2013). Motivation may mitigate this relationship as increasing motivation may increase the utility of the task, reducing the strength of the fatigue signal and maintaining the expenditure of LE.

To better understand the role of experimental context on the relationship between motivation, listening demands and listening effort and to get a sense for the generalisability of results, we carried out two highly similar studies in an online and in a laboratory setting. This was borne out of the observation that there has been a recent surge in interest in performing experiments online (Hartshorne et al., 2019; Anwyl-Irvine et al., 2020; Backx et al., 2020; Shapiro et al., 2020; Bianco et al., 2021; Zhao et al., 2022). Online experiments can be used when access to laboratory research is restricted, when rapid data collection is needed and when access to a larger and more varied group of participants is required (Casey et al., 2017; Bianco et al., 2021). However, potential disadvantages include reduced experimenter control over the participants’ environment (e.g., poor quality headphones/speakers, noisy backgrounds) and participant engagement (Clifford and Jerit, 2014; Chandler and Paolacci, 2017; Zhao et al., 2022). This may account for poorer participant performance when a speech-in-noise task (the Co-ordinate Response Matrix) was completed online versus in the laboratory (Bianco et al., 2021). The potential for reduced participant engagement is particularly relevant for tasks involving effortful listening, as motivation is posited to be a key factor in determining the decision to continue listening in challenging circumstances (Pichora-Fuller et al., 2016). However, studies have not necessarily shown that online participants were less engaged. On the contrary, some studies have shown online participants to be more attentive than laboratory participants (Hauser and Schwarz, 2016).

### Study aims

The current study investigated how the opposing effects of driving (evaluative threat and resource availability) and restraining (listening demands) forces influenced LE in a speech recognition task. We used vocoded stimuli to manipulate listening demands, which have been shown to be effective in eliciting LE both when measured subjectively and objectively (McMahon et al., 2016; Winn, 2016; Winn and Moore, 2018). Speech stimuli were degraded using tone vocoders to create moderate and high listening demand conditions, as in Carolan et al. (2021).

To manipulate motivation with evaluative threat, we used a mild deception by informing participants that they needed to score above a certain threshold for their results to be usable. We assessed resource availability by means of auditory verbal working memory, because the Ease of Language Understanding model identifies working memory as a key mechanism for speech perception under challenging listening conditions (Rönnberg et al., 2019). We assessed auditory working memory by measuring performance on the backwards digit span test (Woods et al., 2011), a measure that has been shown to be a significant predictor of performance in the same speech recognition task as used in the present study (i.e., tone-vocoded speech, intelligibility manipulated through prior exposure) (Carolan et al., 2021).

We asked the following questions regarding a speech recognition task:

1. Does motivation (evaluative threat) interact with listening demands to affect task performance, LE, and tiredness?
2. Is resource availability associated with performance, LE and self-rated tiredness?

Based on previous work we predicted the following main effects:

- Higher listening demands would lead to decreased performance accuracy, longer RTs (suggesting greater LE) and increased self-rated work.
- Higher motivation would lead to decreased self-rated tiredness.
- Better working memory would be associated with better performance accuracy and less LE.

Furthermore, we predicted the following interactions between motivation and listening demands:

- Moderate listening demands*motivation: performance accuracy, RTs and self-rated work would be unaffected by differences in motivation.
- High listening demands*motivation: high motivation would lead to higher performance accuracy, lower self-rated work and shorter RTs (suggesting less effort) than low motivation in the absence of evaluative threat.

Based on previous studies (Hauser and Schwarz, 2016; Harrison and Müllensiefen, 2018; Bianco et al., 2021), we expected the experimental setting (online/laboratory) to impact results and therefore, asked:

3 What impact does experimental setting (online/laboratory) have on performance and indices of LE in a speech recognition task?

We expected similar LE (RTs, self-rated work) in the online and laboratory settings but reduced performance accuracy in the online setting, as participants may be more prone to distractions in this less controlled environment.

## Laboratory experiment

### Methods

#### Participants

Thirty-seven (18 female, 19 male) normal-hearing native speakers of English between the ages of 18 to 35 years old (median = 23) participated in the laboratory experiment. Eligible participants had normal or corrected-to-normal vision and no previous neurological issues or speech problems.

The required sample size of 37 was calculated using the Simr software package (Green and MacLeod, 2016) in R (R Core Team, 2018), which estimates sample size based on mixed-effects analyses. The power calculation used the effect size of financial reward on self-rated work reported in Carolan et al. (2021). The calculations aimed to find the sample size required to detect a slope of 0.07, equivalent to a medium effect (Cohen’s *f^2^* = 0.15) of motivation on the correct response rate and self-rated work in the speech recognition task, with 80% power where α = 0.05 using 1,000 simulations.

The University of Manchester Research Ethics Committee reviewed and approved the study (approval number: 2021-12,598-19975) and we pre-registered the protocol with the Open Science Framework.^1^

### Materials

#### Speech recognition task: stimuli

**Figure 1:**
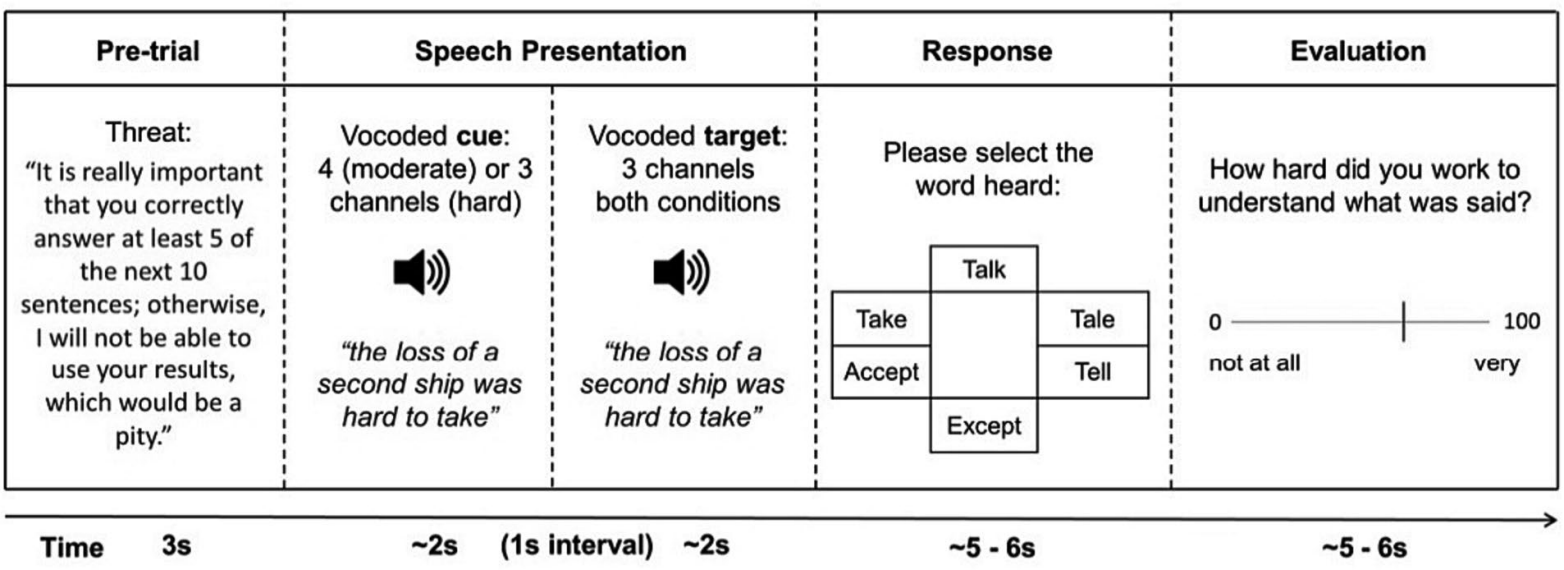
Diagram of a typical trial with evaluative threat. In trials without evaluative threat, the pre-trial screen informed participants that “*I can use your results for this next group of 10 sentences as long as you answer at least a few of them correctly*”.

The speech recognition task required participants to listen carefully to vocoded sentence stimuli and select, using a mouse, a key word contained at the beginning, middle or end of a sentence from among several foils on a visual word grid (see Figure 1). The speech recognition task was based on that used in Carolan et al. (2021). Ninety-five of the shortest duration Harvard IEEE sentences (Rothauser et al., 1969) spoken by a male speaker, were used in the speech recognition task, including 15 sentences that were used during training. Tone vocoders with the frequency of each vocoder band logarithmically spaced between 80 and 8,000 Hz were used to modify speech intelligibility in a controlled fashion (Drullman et al., 1994; Shannon et al., 1995). See Carolan et al. (2021) for further details of the vocoder methods.

Listening demands were manipulated with 2- and 3-band vocoders in order to best match overall speech perception accuracy, as per Carolan et al. (2021): They showed 2-band vocoded speech to have a mean correct response rate of 50.2 RAU and 3-band vocoded speech to have a mean correct response rate of 69.6 RAU.

#### Procedures

All participants had bilateral normal hearing according to pure-tone audiometry (PTA) hearing thresholds (≤20 dB HL for test frequencies of 250, 500, 1,000, 2,000, 4,000 and 8,000 Hz; British Society of Audiology, 2018). Participants reported no recent ear infections or surgery, previous neurological issues or speech problems.

Participants completed all tasks in a single testing session which lasted around 1 h. Participants were paid £15 for participation. For the speech recognition task and backwards digit span task, participants were seated in a sound-attenuated booth facing a computer monitor. During each trial, vocoded sentences were presented diotically at a fixed level of 65 dB(A) via loud speakers at ±45° azimuth.

Figure 1 shows an outline of a trial in the speech recognition task. Each trial consisted of two presentations of a vocoded speech sentence. In the moderate listening demands condition, the first presentation (“cue”) of the sentence was processed to produce a moderate level of intelligibility (3-band vocoder) followed by a second presentation (“target”) processed for low speech intelligibility (2-band vocoder). In the high listening demands condition, the intelligibility was always low, i.e., both “cue” and “target” sentences were processed to produce low speech intelligibility (2-band vocoder). The “target” sentence was always processed with a 2-band vocoder and was therefore physically identical in both the high and moderate listening demands conditions.

Motivation was manipulated using evaluative threat. Prior to blocks with evaluative threat, participants were informed via text on the computer screen that: “*It is really important that you correctly answer at least 5 of the next trials, otherwise I will not be able to use your results, which would be a pity*.” The wording of this manipulation was based on Zekveld et al. (2019). Prior to blocks without evaluative threat, participants were informed “*I can use the data collected in this next batch of sentences as long as you answer a few correctly*.”

The operationalisation of motivation in this study involved a mild deception because, in the condition with evaluative threat, participants were led to believe that they needed to score above a certain threshold for their results to be usable, even though this was untrue and all results were reported and included in the analyses. After completing the speech recognition task, we debriefed participants on why mild deception was necessary.

Prior to beginning the test blocks, participants completed a practice block consisting of 15 trials: 5 trials using clear (unprocessed) speech to familiarise participants with the procedure, 5 trials using 3-band vocoded speech for the cue sentence (i.e., moderate listening demands) and then 5 trials using 2-band vocoded speech for the cue sentence (i.e., high listening demands). After participants had completed the practice sentences, they were informed that “*Based on your performance on the practice sentences, you should be able to give the correct answer for around 5 out of every 10 sentences*.”

After the practice block, 80 test trials were presented in 8 blocks of 10 trials each. There were four blocks with evaluative threat and four blocks without evaluative threat, presented in a random order. Each block consisted of 5 trials with high listening demands and 5 trials with moderate listening demands, presented in a random order.

### Outcome measures

#### Correct response rate and RTs

The percentage of correct responses (correct response rate) as well as the mean response time (RT), inclusive of incorrect trials, were measured.

Speech intelligibility was assessed using the same method as Carolan et al. (2021). A test word was randomly selected from either the beginning, middle or end of the sentence to ensure that participants needed to listen to the entire stimulus. Participants were asked to select, using a mouse, which word they had heard within the preceding sentence from among five foils presented as a 6-word visual grid (see Figure 1). The visual grid was arranged so that each option was roughly equidistant from the centre of the screen. Participants were asked to return the mouse cursor to the middle of the screen during stimulus presentation. The location of the test word varied randomly within the 6-word grid, with an equal chance of the test word appearing in any of the 6 positions. Foils were either phonologically or semantically related to the test word or other foils. For example, for the sentence “tend the sheep while the dog wanders” and the test word “sheep,” we used foils phonologically related to “sheep” (e.g., “sheet,” “shoot”), a semantic foil for “sheep” (e.g., “cattle”) and foils which were phonologically or semantically related to other foils (e.g., “kettle,” “blanket”). An online rhyming dictionary, rhymezone.com, was used to select phonologically and semantically related foils. For a given word, the website provides a list of similar-sounding and semantically-related words. The relatedness of each word to the original word is rated by the developers between 0 and 100. We chose foils that were rated 90 or more out of 100.

Participants were requested to respond as accurately and quickly as possible. To ensure only evaluative threat influenced motivation, no performance feedback was provided during the speech recognition task.

#### Subjective ratings

Self-rated work: After each trial in the speech recognition task (see Figure 1), participants were asked *“How hard did you work to understand what was said?”* to measure subjective LE.

Self-rated tiredness: After every 10^th^ trial, participants were asked *“How tired of listening are you?”*

Participants were asked to provide subjective ratings (0–100%) using a visual scale. We gauged subjective ratings of LE and self-rated tiredness using questions with similar wording to Picou and Ricketts (2014, 2018) who in turn had based these questions on items from the Speech, Spatial and Qualities Hearing Scale (Gatehouse and Noble, 2004).

#### Working memory task

After finishing the speech recognition task, participants completed the backwards digit span test.

In a backwards digit span task, a series of digits was presented and participants were asked to recall the digits in reverse order, as described in Woods et al. (2011). Two practice trials were presented to familiarise participants with the task prior to the main assessment. In the main assessment, participants were initially presented with a 2-digit sequence. The sequence length was subsequently increased by one if the participant recalled the sequence correctly. If an incorrect response was given, the participant was given two more attempts with new sequences of the same length. After three unsuccessful attempts, the sequence length was decreased by one. The longest sequence correctly repeated after 14 trials corresponded to a participant’s backwards digit span.

### Data analyses

Correct response rates, self-rated work and self-rated tiredness were converted to rationalized arcsine units (RAU) (Studebaker, 1985) prior to statistical analysis. A RAU transform was performed to ensure that the data were suitable for statistical analysis; often percentage data are not normally distributed around the mean, or have variances correlated with the mean (Studebaker, 1985). Outliers (further than three standard deviations from the mean for each participant) were removed from the RT data (Picou et al., 2017). A log_10_ transformation was applied to RTs to ensure data met the assumption of normality for parametric statistics.

Linear mixed models were fitted in R (R Core Team, 2018) to investigate predictors of the correct response rate and LE outcomes. Full models included fixed effects for listening demands (high/moderate listening demands), motivation (with/without evaluative threat), listening demands*motivation and backwards digit span score. Participants were modelled as random intercepts.

In all cases the final model was determined using a backward stepwise procedure, as suggested by Pinheiro and Bates (2000). Following this procedure, the original full model, which included all main effects and interactions, was pruned so that the final model included higher-level interaction terms only if they improved model fit. Model fit was estimated by the.

Akaike Information Criterion (AIC), an index of the relative fit of a mixed model. The AIC values of the model with and without a particular term were compared. If the simpler model led to a reduction in AIC, it was adopted. If the simpler model led to an increase in AIC, the AIC values of the two models were compared using an ANOVA to compare both model fits. If the pruned model was not significantly worse, it was brought forward as the new base model. Only when the more complex model led to a significantly better fit was the higher-level interaction kept in the model. The principle of marginality was observed such that if a higher-level interaction was kept in the model, the nested lower-level (subordinate) interactions were also retained without testing. For example, if A × B × C was kept in the model, the model also included A × B, A × C, and B × C. Main effects were always kept in the model. Fixed effects were successively pruned using this method. The final model was established when no further pruning could be carried out without resulting in a significantly worse fit. The statistical significance of the effects was determined using type II ANOVA within the lme package (R Core Team, 2018).

Since self-ratings of tiredness were collected at the end of each motivation block, rather than on a trial-by-trial basis, a paired sample *t*-test was used to compare mean self-ratings of tiredness for conditions with/without evaluative threat conditions.

### Results

**Figure 2:**
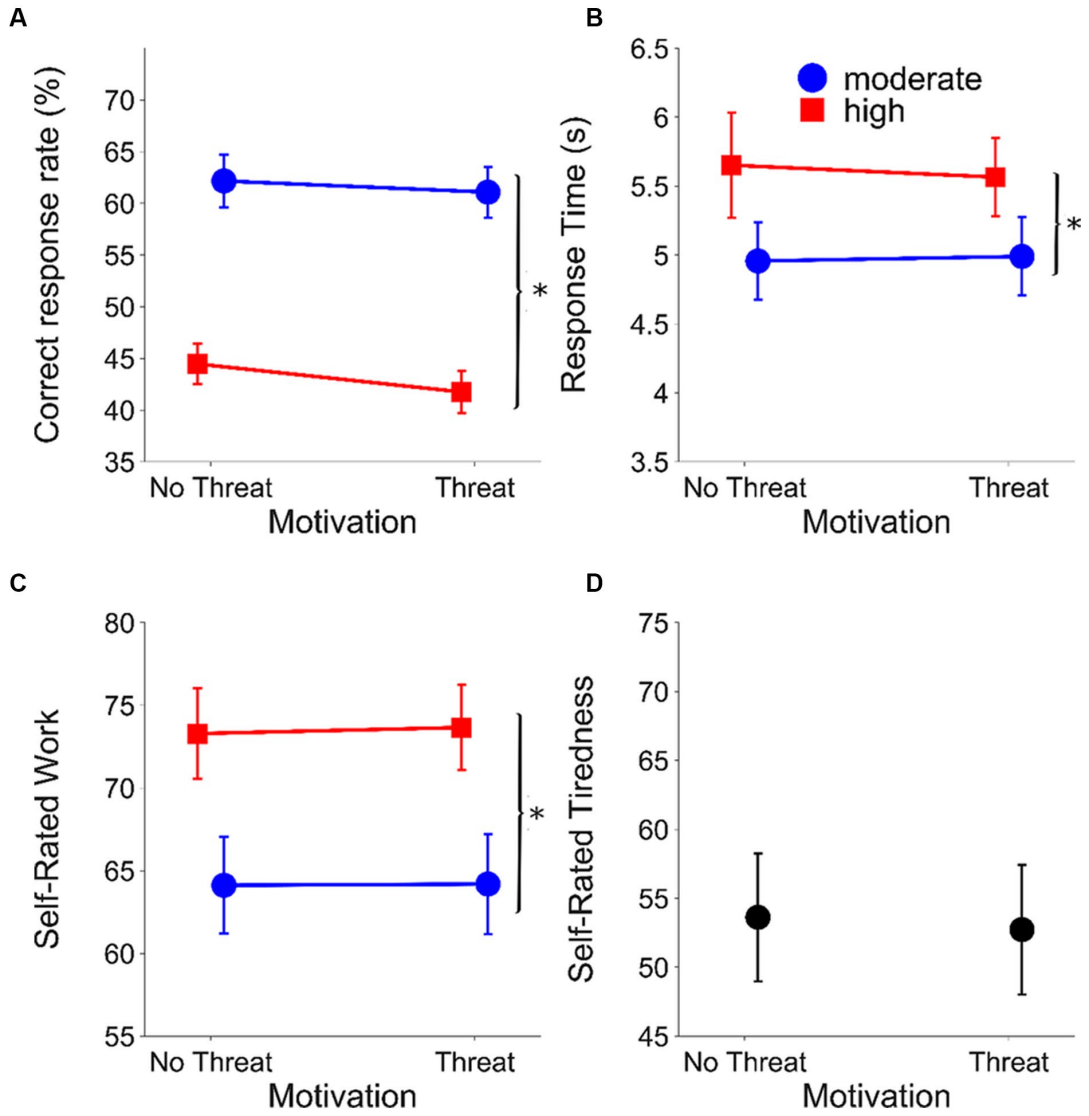
Results from the laboratory setting. **(A)** Correct response rate (%), **(B)** mean RTs (s), **(C)** mean self-rated work (%) and **(D)** mean self-rated tiredness (%) as a function of evaluative threat for the speech recognition task (laboratory experiment) (**p < 0.001; *p < 0.05). Circles (blue) represent the moderate listening demands condition; squares (red) represent the high listening demands condition. Error bars represent ±1 standard error of the mean. Results within motivation conditions are offset to aid visualisation.

Figure 2 shows the effects of listening demands and motivation on performance (correct response rate) and indices of LE/self-rated tiredness. Table 1 shows the results of the final mixed models for each outcome measure after pruning.

**Table 1:** Summary of final models for the laboratory study.

Correct response rate					
Fixed effects					
	Est/Beta	SE	95% CI	*t*	*p*
Intercept	0.250	0.032	0.184, 0.308	7.805	<0.001**
Demands	0.185	0.018	0.150, 0.220	10.423	<0.001**
Random effects					
		Variance		SD	
Participant (intercept)		0.008		0.087	
Model fit					
R^2^		Marginal		Conditional	
		0.065		0.034	
Model equation: Correct response rate ~ 1 + Difficulty + (1| Participant)					

RT					
Fixed effects					
	Est/Beta	SE	95% CI	*t*	*p*
Intercept	0.723	0.024	0.676, 0.771	29.688	<0.001**
Demands	−0.070	0.009	−0.088, −0.051	−7.433	<0.001**
Random effects					
		Variance		SD	
Participant (intercept)		0.014		0.118	
Model fit					
R^2^		Marginal		Conditional	
		0.184		0.016	
Model equation: RT ~1 + Demands + (1| Participant)					

Self-rated work					
Fixed effects					
	Est/Beta	SE	95% CI	*t*	*p*
Intercept	87.480	3.444	80.729, 94.232	25.397	<0.001**
Demands	−10.789	0.818	−12.392, −9.186	−13.194	<0.001**
Random effects					
		Variance		SD	
Participant (intercept)		376.903		19.414	
Model fit					
R^2^		Marginal		Conditional	
		0.451		0.032	
Model equation: Self-rated work ~1 + Demands + (1| Participant)					

**p < 0.001; *p < 0.05.

The final model for the correct response rate included a significant fixed effect of listening demands (*β* = 0.19, SE = 0.02, *t* = 10.42, *p* < 0.001). The correct response rate was significantly higher in the moderate listening demands condition compared to the high listening demands condition (moderate: mean = 0.62 RAU, SEM = 0.013; high: mean = 0.43 RAU, SEM = 0.013).

For RT, the final model included a significant fixed effect of listening demands (*β* = −0.07, SE = 0.01, *t* = −7.43, *p* < 0.001), with longer RTs under higher listening demands (moderate: mean = 0.58 log_10_(s), SEM = 0.007; high: mean = 0.65 log_10_(s), SEM = 0.007).

The final model for self-rated work included a significant fixed effect of listening demands (*β* = −10.79, SE = 0.82, *t* = −13.19, *p* < 0.001). Self-rated work was greater in the high compared to moderate listening demands conditions (moderate: mean = 65.90 RAU, SEM = 0.81; high: mean = 76.69 RAU, SEM = 0.72).

The effect of motivation on self-rated tiredness was not significant (*t*(36) = −0.352, *p* = 0.727) (with evaluative threat mean = 52.78, SEM: 4.65; without evaluative threat mean = 53.42, SEM: 4.48).

## Online experiment

### Methods

#### Participants

Thirty-seven (26 female, 11 male) normal-hearing listeners between the ages of 18 to 36 years old (median = 25 years) participated in the online experiment. They were recruited via the website Prolific.co and paid via the online platform at Prolific’s standard rate of £6.50 per hour. Participants who completed the laboratory experiment were excluded. Eligible participants were self-reported native speakers of English and had self-reported normal or corrected-to-normal vision and no previous neurological issues or speech problems.

To check hearing status, participants undertook the HearWHO via a smartphone app (https://www.who.int/health-topics/hearing-loss/hearwho). The HearWHO is an online digits-in-nose test designed to allow individuals to check and monitor their hearing status. The HearWHO is a pass/fail screening test. A HearWHO score of 50 or more was considered to represent a “pass” i.e. “normal hearing.” The HearWHO app advises individuals scoring less than 50 to seek professional testing/advice regarding a possible hearing loss.

The University of Manchester Research Ethics Committee reviewed and approved the study (approval number: 2021-10,372-17457) and we pre-registered the protocol with the Open Science Framework.^2^

### Materials

The speech recognition task completed in the online experiment was largely comparable to that used in the laboratory experiment (see Figure 1 and *Laboratory experiment: Methods* for details).

#### Speech recognition task: stimuli

The number of vocoder bands used in the online setting was set to 3 (high listening demands condition) and 4 (moderate listening demands condition), which was higher than the number used in the laboratory-based experiment: i.e. 2 for the high listening demands condition and 3 for the moderate listening demands condition. This modification was necessary to control for difficulty between paradigms. Our pilot data indicated a much lower correct response rate in the high listening demands condition in the online study (29% correct), compared to the laboratory-based results reported above. Using 3- and 4-band stimuli resulted in correct response rates more closely aligned with the performance of the laboratory-based study.

#### Procedures

Participants were instructed to wear headphones throughout the study session.

##### Headphone check

All participants completed a headphone check designed by Woods et al. (2017). In this task, participants must correctly detect an acoustic target in an intensity-discrimination task. This task involved the use of tones which sometimes had a phase difference of 180° between stereo channels, “anti-phase” tones. Participants who are not using headphones tend to make incorrect intensity discriminations to “anti-phase” tones, as these are heavily attenuated when played through speakers but not through headphones. All participants answered at least 5 out of 6 trials correctly, indicating that they were using headphones appropriately. Participants were instructed to play the stimuli at a comfortable listening level. An additional check for presentation level was not carried out.

##### Speech recognition task

Participants completed a practice block of 15 trials. This included 5 trials with clear (unprocessed) speech, 5 trials with 4-band vocoded speech for the cue sentence (i.e., moderate listening demands) and 5 trials using 3-band vocoded speech for the cue sentence (i.e., high listening demands). After participants had completed the practice sentences, they were informed that “*Based on your performance on the practice sentences, you should be able to give the correct answer for around 5 out of every 10 sentences*.” 80 test trials were then presented in the same manner as the laboratory experiment (see Laboratory Experiment: Methods for further details).

##### Data analyses

The sample size of *N* = 37 provided >80% adequate power to detect a slope of 0.07, which is equivalent to a medium-sized effect (Cohen’s *f^2^* = 0.15) of motivation on correct response rates and self-rated work in the speech recognition task (see *Laboratory Experiment: Methods* further details).

Data were pre-processed before statistical analyses in the same way as described for the laboratory experiment (see *Laboratory Experiment: Methods: Data Analysis*). Full linear mixed models consisted of fixed effects for listening demands, motivation (with/without evaluative threat) and listening demands*evaluative threat, and working memory (backwards digit span scores). Participants were coded as random intercepts. Full models were pruned to find the best-fitting model in order to investigate predictors of the correct response rate and LE outcomes (see *Laboratory Experiment: Methods* for further details of the pruning process). A paired sample *t*-test was used to compare mean ratings of self-rated tiredness for conditions with/without evaluative threat.

We compared results of the online and laboratory experiments by fitting an additional linear mixed model for each outcome measure, which included setting (laboratory/online) as an additional fixed effect. Therefore, these models included fixed effects for listening demands, motivation, working memory, and listening demands*motivation, setting (i.e., online or laboratory), listening demands*setting, motivation*setting and listening demands*motivation*setting, as well as participants as random intercepts. Again, these models were pruned following the procedure above.

### Results

**Figure 3:**
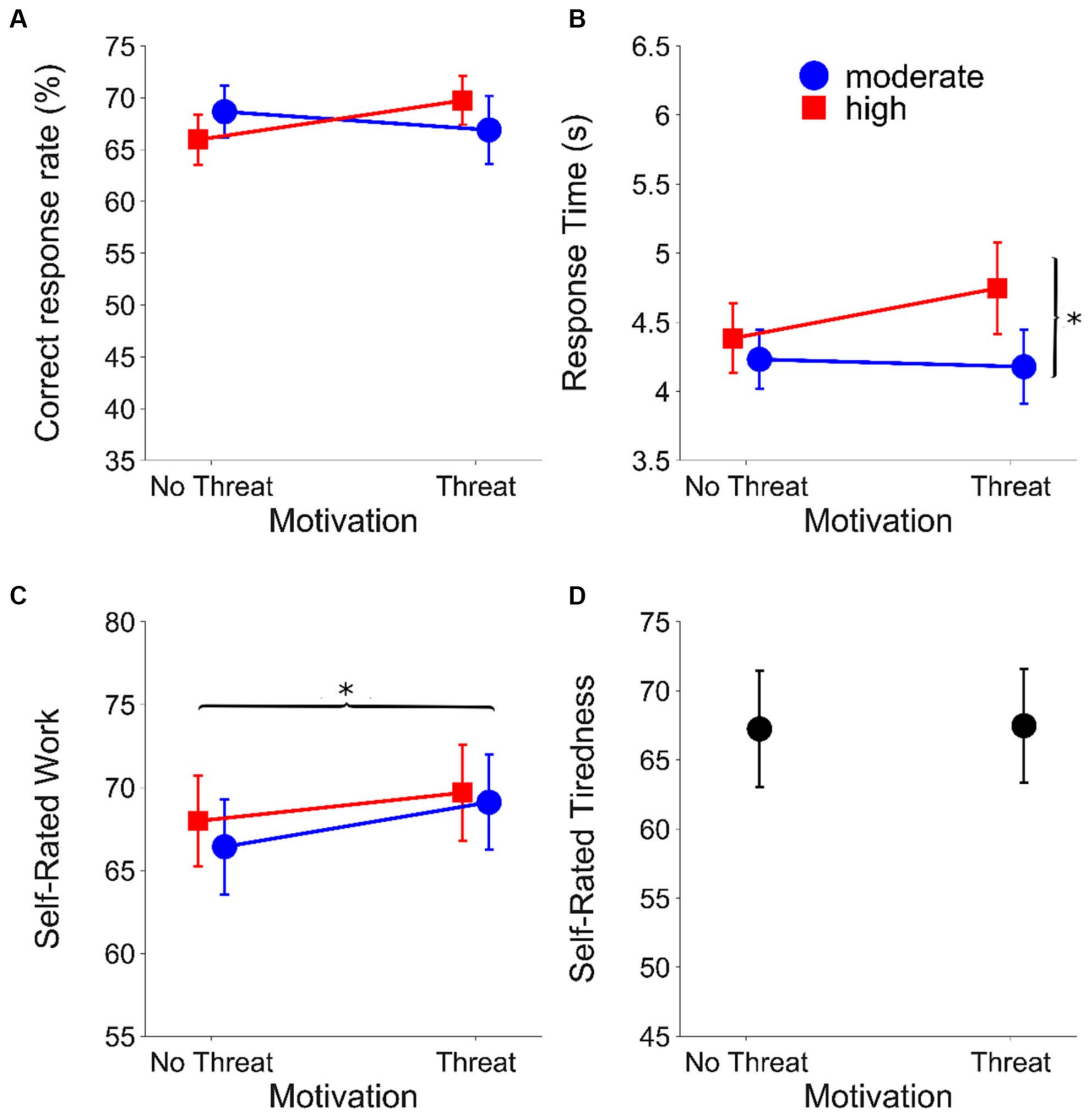
Results from the online setting. **(A)** Correct response rate (%), **(B)** mean RTs (s), **(C)** mean self-rated work (%) and **(D)** mean self-rated tiredness (%) as a function of evaluative threat for the speech recognition task (online experiment) (**p < 0.001; *p < 0.05). Circles (blue) represent the moderate listening demands condition; squares (red) represent the high listening demands condition. Error bars represent ±1 standard error of the mean. Results within motivation conditions are offset to aid visualisation.

Figure 3 shows the results of the online speech recognition task for each outcome measure (correct response rate, RT, self-rated work and self-rated tiredness). Table 2 shows the statistical parameters for the final mixed models for each outcome measure after pruning, with the predictor variables coded as fixed effects.

**Table 2:** Summary of final models for the online study.

Correct response rate					
Fixed effects					
	Est/Beta	SE	95% CI	*t*	*p*
Intercept	0.698	0.018	0.664, 0.733	39.604	<0.001**
Random effects					
		Variance		SD	
Participant (intercept)		0.008		0.088	
Model fit					
R^2^		Marginal		Conditional	
		0.036		<0.001	
Model equation: Correct response rate ~ 1 + (1| Participant)					

RT					
Fixed effects					
	Est/Beta	SE	95% CI	*t*	*p*
Intercept	0.345	0.115	0.120, 0.571	2.998	0.003*
Demands	−0.028	0.010	−0.047, −0.008	−2.769	0.006*
Backwards Digit Span	0.034	0.017	0.001, 0.035	2.059	0.048*
Random effects					
		Variance		SD	
Participant (intercept)		0.009		0.095	
Model fit					
R^2^		Marginal		Conditional	
		0.139		0.019	
Model equation: RT ~1 + Demands + Backwards Digit Span + (1| Participant)					

Self-rated work					
Fixed effects					
	Est/Beta	SE	95% CI	*t*	*p*
Intercept	70.013	3.460	63.233, 76.794	20.236	<0.001**
Demands	−1.278	0.929	−3.100, 0.542	−1.376	0.169
Motivation	2.491	0.929	0.671, 4.311	2.682	0.007*
Random effects					
		Variance		SD	
Participant (intercept)		316.377		17.787	
Model fit					
R^2^		Marginal		Conditional	
		0.363		0.002	
Model equation: Self-rated work ~1 + Demands + Motivation + (1| Participant)					

**p < 0.001; *p < 0.05.

The final model for the correct response rate did not include any of the predictors (i.e., listening demands, motivation, listening demands*motivation or backwards digit span). Thus there were no main effects or interactions on this outcome measure.

For RT, the final model included significant fixed effects of listening demands (*β* = −0.03, *t = −2.77,* SE = 0.01, *p* = 0.006) and backwards digit span (*β* = 0.03, *t* = 2.06, SE: 0.02, *p* = 0.048). RTs were longer under higher listening demands (moderate: mean = 0.53 log_10_(s), SEM = 0.007; high: mean = 0.56 log_10_(s), SEM = 0.007). Greater resource availability (larger backwards digit span) was associated with longer RTs.

The final model for self-rated work included significant fixed effects of motivation (*β* = 2.49, *t* = 2.68, SE = 0.93, *p* = 0.007) There was a significant increase in self-rated work under conditions with evaluative threat (without evaluative threat: mean = 69.61 RAU, SEM = 0.80; with evaluative threat: mean = 72.31 RAU, SEM = 0.79). The final model also included a non-significant fixed effect of listening demands.

Motivation did not affect self-rated tiredness (with evaluative threat mean = 67.98, SEM = 4.37; without evaluative threat mean = 67.76, SEM = 4.50) (*t*(36) = 0.147, *p* = 0.884).

### Comparison of online and laboratory results

**Figure 4:**
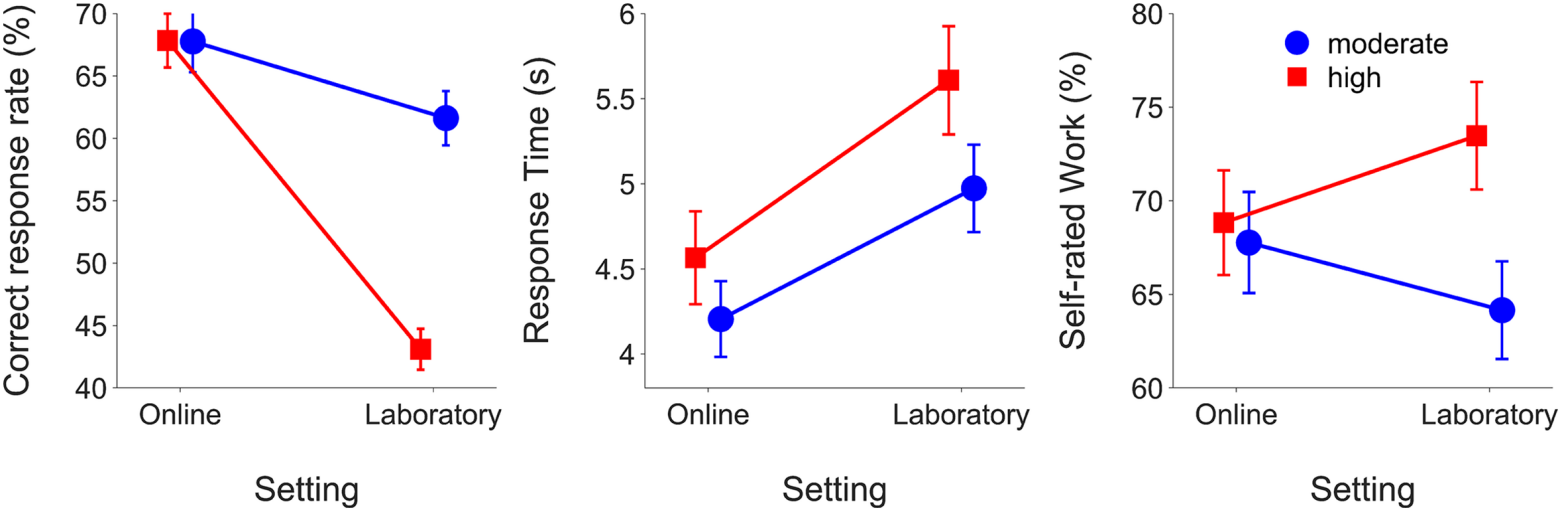
Means and SEMs for outcomes from the online experiment and the laboratory experiment grouped by listening demands condition.

Table 3 shows the results of final mixed models comparing the online and laboratory experiments after pruning. The final model for the correct response rate included significant fixed effects for listening demands (*β* = −0.19, SE = 0.04, *t* = −9.54, *p* < 0.001), setting (*β* = −0.43, SE = 0.05, *t* = −9.54, *p* < 0.001) and listening demands*setting (*β* = 0.19, SE = 0.02, *t* = 7.64, *p* < 0.001). When results were collapsed across both experiments, correct response rates were higher in the moderate compared to high demands listening demands condition (moderate: mean = 0.65 RAU, SEM = 0.009; high: mean = 0.55 RAU, SEM = 0.009). The correct response rates were higher in the online experiment compared to the laboratory experiment (online: mean = 0.68 RAU, SEM = 0.009; laboratory: mean = 0.52 RAU, SEM = 0.009). Figure 4 shows means and SEM for outcomes from the online and laboratory experiments. Listening demands were a significant fixed effect in the final correct response rate model for laboratory experiment but not for the correct response rate model for the online experiment.

**Table 3:** Summary of models comparing results from the online and laboratory experiments.

Correct response rate					
Fixed effects					
	Est/Beta	SE	95% CI	*t*	*p*
Intercept	1.112	0.072	0.971, 1.252	15.493	<0.001**
Demands	−0.186	0.038	−0.262, −0.111	−9.540	<0.001**
Setting	−0.433	0.045	−0.524, −0.343	−9.540	<0.001**
Demands:Setting	0.186	0.024	0.138, 0.233	7.638	<0.001**
Random effects					
		Variance		SD	
Participant (intercept)		0.011		0.104	
Model fit					
R^2^		Marginal		Conditional	
		0.088		0.043	
Model equation: Correct response rate ~ 1 + Demands + Setting + Demands:Setting + (1| Participant)					

RT					
Fixed effects					
	Est/Beta	SE	95% CI	*t*	*p*
Intercept	0.475	0.042	0.392, 0.557	11.285	<0.001**
Setting	0.122	0.027	0.068, 0.177	4.469	<0.001**
Demands:Setting	−0.033	0.004	−0.041, −0.025	−7.950	<0.001**
Random effects					
		Variance		SD	
Participant (intercept)		0.012		0.111	
Model fit					
R^2^		Marginal		Conditional	
		0.173		0.026	
Model equation: RT ~1 + Setting + Demands:Setting + (1| Participant)					

Self-rated work					
Fixed effects					
	Est/Beta	SE	95% CI	*t*	*p*
Intercept	55.762	7.860	40.361, 71.163	7.094	<0.001**
Demands	8.203	1.876	4.527, 8.203	4.373	<0.001**
Motivation	5.120	1.876	1.445, 8.795	2.729	0.006*
Setting	15.790	4.971	5.885, 25.695	3.176	0.002*
Demands:Setting	−9.496	1.186	−11.820, −7.171	−8.004	<0.001**
Motivation:Setting	−2.421	1.186	−4.754, −0.097	−2.041	0.041*
Random effects					
		Variance		SD	
Participant (intercept)		385.219		19.627	
Model fit					
R^2^		Marginal		Conditional	
		0.435		0.017	
Model equation: Self-rated work ~1 + Demands + Motivation + Setting + Demands:Setting + Motivation:Setting + (1| Participant)					

The experimental context (online/laboratory) was coded as “Setting” (**p < 0.001; *p < 0.05).

For RTs, the final model included significant fixed effects for setting (*β* = 0.12, SE = 0.03, *t* = 4.47, *p* < 0.001) and listening demands*setting (*β* = −0.03, SE <0.01, *t* = −7.95, *p* < 0.001).

RTs were longer when listening demands were high compared to moderate (moderate: mean = 0.56 log_10_(s), SEM = 0.005; high: mean = 0.61 log_10_(s), SEM = 0.005). RTs were longer overall in the laboratory compared to online (online: mean = 0.55 log_10_(s), SEM = 0.005; laboratory: mean = 0.62 log_10_(s), SEM = 0.005). Figure 4 shows RTs (means and SEMs) in the online and laboratory experiments. RTs slowed to a greater extent with higher listening demands in the laboratory versus online.

The final model for self-rated work included significant fixed effects for listening demands (*β* = 8.20, SE = 1.88, *t* = 4.37, *p* < 0.001), motivation (*β* = 5.12, SE = 1.88, *t* = 2.73, *p* = 0.006), setting (*β* = 15.79, SE = 4.97, *t* = 3.18, *p* = 0.002), listening demands*setting (*β* = −9.50, SE = 1.19, *t* = −8.00, *p* < 0.001) and motivation*setting (*β* = −2.42, SE = 1.19, *t* = −2.04, *p* = 0.041).

**Figure 5:**
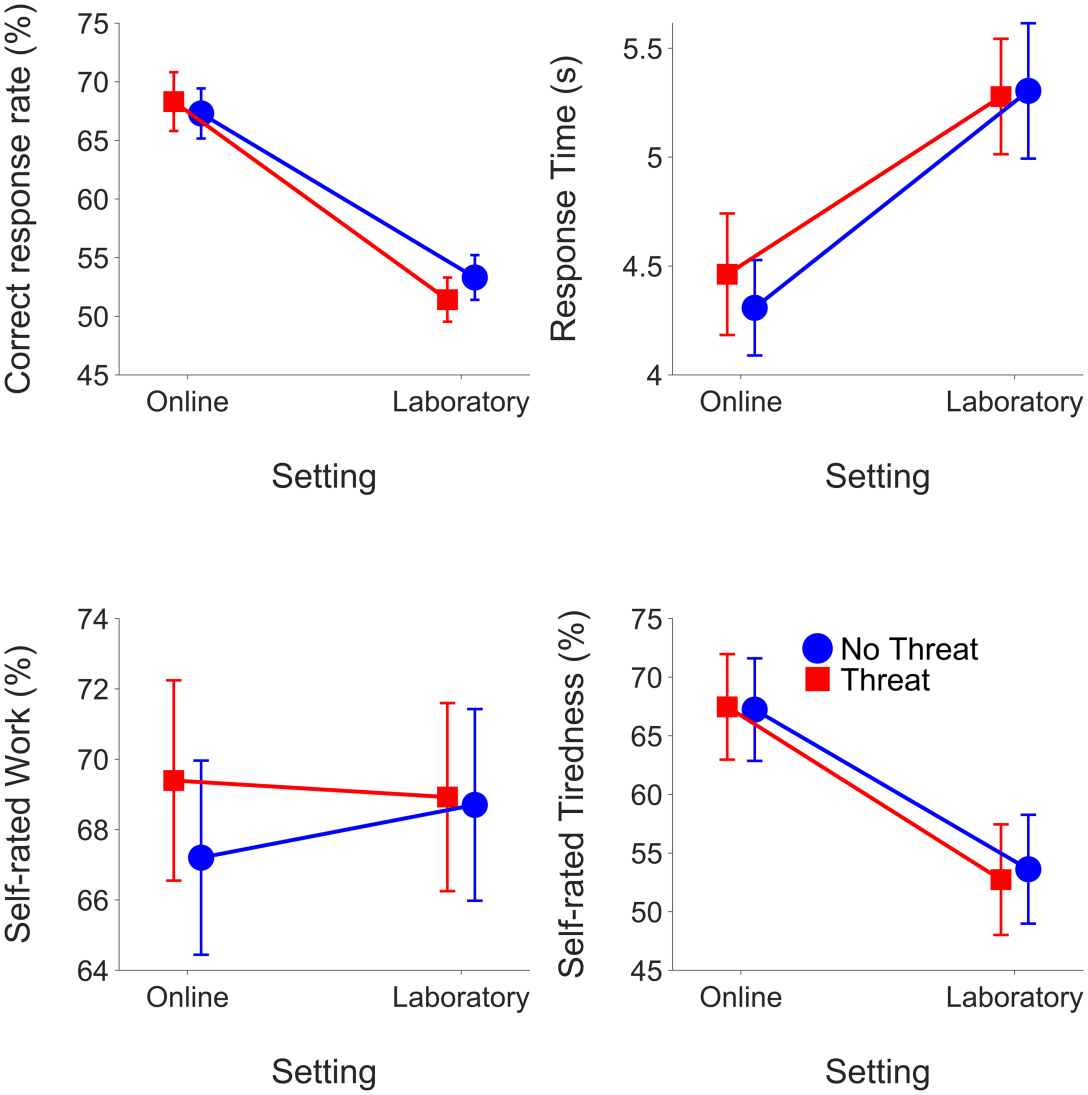
Means and SEMs for outcomes from the online experiment and laboratory experiment grouped by motivation condition.

Self-rated work was greater in the high compared to moderate listening demands condition (moderate: mean = 68.11 RAU, SEM = 0.57; high: mean = 74.15 RAU, SEM = 0.54). Self-rated work was also greater in conditions with evaluative threat (without evaluative threat: mean = 70.39 RAU, SEM = 0.56; with evaluative threat: mean = 71.87 RAU, SEM = 0.55). High listening demands increased self-rated work to a greater extent in the laboratory compared to the online setting. Figure 5 shows the effects of motivation on the means and SEM for each LE outcome. Evaluative threat increased self-rated work to a greater extent in the online than in the laboratory study.

Table 4 shows means, standard deviations and ranges for the backwards digit span task carried out as part of the online and laboratory experiments.

**Table 4:** Mean, standard deviation and range for the backwards digit span task carried out online and in a laboratory setting.

	Mean	Standard deviation	Range
Backwards digit span (online)	7.16	1.69	4–12
Backwards digit span (laboratory)	6.86	1.03	5–8

## Discussion

We investigated the relationship between restraining factors such as listening demands, and driving factors such as motivation and resource availability for LE and tiredness in a speech recognition task in both online and laboratory settings. Listening demands were manipulated by varying the extent of degradation of vocoded speech presented to participants. Motivation was manipulated using evaluative threat. Resource availability was measured as backwards digit span score. Outcome measures were performance accuracy of perception of vocoded sentences and indices of LE and self-rated tiredness. These variables were chosen to test theories predicting a greater influence of motivation on listening effort and tiredness under high, compared to low, listening demands (Brehm and Self, 1989; Kruglanski et al., 2012; Hockey, 2013; Pichora-Fuller et al., 2016). To examine the effects of experimental setting we carried out the speech recognition task both in a controlled laboratory setting and online.

### Does motivation interact with listening demands to affect task performance (performance accuracy), indices of LE (RTs, self-rated work) and tiredness in a speech recognition task?

Motivation was predicted to have a greater influence on effort exertion under high (but not impossible) compared to low listening demands, resulting in an interaction effect. In the laboratory experiment, main effects of listening demands on correct response rates and indices of LE were found, with large effects measured with all LE outcomes. Higher listening demands led to a significant reduction in correct response rates, slower RTs and higher self-rated work. This replicates our previous laboratory study using the same task (Carolan et al., 2021). Our prediction of an interaction between listening demands and motivation was not supported. There were also no main effects of motivation for any of the outcomes. This is in contrast with previous studies using subjective and behavioural measures that report an increase in LE with evaluative threat (Carrillo-de-la-Pena and Cadaveira, 2000; Picou and Ricketts, 2014; Zekveld et al., 2019). The evaluative threat manipulation in the laboratory experiment, which was displayed on the computer screen, may not have been effective in increasing motivation. In Zekveld et al. (2019), the researchers stopped the experiment, entered the testing room and communicated the evaluative threat verbally. This procedure was repeated twice. In addition, in Zekveld et al. (2019), participants received false feedback on their performance to further motivate them to exert LE. Picou and Ricketts (2014), who also found an increase in LE with evaluative threat, tested participants verbally, gave feedback and used longer stimuli, which may have heightened anxiety. Using more effective methods of motivating participants based on evaluative threat may have led to significant increases in LE in the present study.

In the online experiment, no significant listening demand by motivation interactions were found for any of the outcomes. These results contrast with Picou and Ricketts (2014) who report increased subjective ratings of LE with evaluative threat under conditions of “hard” but not “easy” listening demands. In the online experiment, performance accuracy was almost identical in the moderate and high listening demands conditions and the predicted effect of greater LE with higher listening demands was found for one LE outcome measure only: RTs. Thus, in the online experiment, there were likely insufficient differences in speech intelligibility between the listening demand conditions to elicit interactive effects. The lack of significant effects of listening demands was unexpected since the laboratory study using the same speech recognition task found large effects of listening demands. This result illustrates the challenges of adapting laboratory-based LE experiments to online settings, where experimenters have less control over participants’ listening environments, the equipment used for listening and participants’ engagement in the listening task.

Despite the null effects of listening demands, and the non-significant interaction between motivation and listening demands, a small main effect of motivation on self-rated work was found in the online experiment. This aligns with previous work finding greater subjective ratings of effort with evaluative threat (Zekveld et al., 2019).

Neither experiment provided evidence in support of the notion that motivation reduces self-rated tiredness in a speech recognition task. The effect of motivation on self-rated tiredness was non-significant in both the laboratory and the online experiment, contradicting the Motivational Control Model (Hockey, 2013) and a previous study finding a reduction in self-rated tiredness when participants are informed of an upcoming test (Picou and Ricketts, 2014). A longer/more demanding speech recognition task may be needed to elicit self-rated tiredness. In Picou and Ricketts (2014), testing lasted “no longer than two hours” (p. 421), whereas here the speech recognition task lasted around 40 min. Additionally, recording self-rated tiredness more frequently than at the end of each block may be necessary to uncover effects of motivation (see McGarrigle et al., 2021).

In summary, in both laboratory and online settings, the interaction between motivation and listening demands on performance accuracy/indices of LE was non-significant.

### Is resource capacity (backwards digit span) associated with performance on a speech recognition task and indices of LE?

Backwards digit span was not a significant predictor in any of the models used to analyse the results for the laboratory experiment, and was only a significant predictor in one mixed model (RTs) in the online experiment. There are a number of potential reasons for these null effects. First, the literature on the association between resource availability and behavioural performance or indices of LE is equivocal, with some studies reporting associations (Zekveld et al., 2012; Wendt et al., 2016; Koelewijn et al., 2021) while others do not (Brown and Strand, 2018; Koelewijn et al., 2018; Hunter, 2021). Second, the lack of predictive effect in the laboratory experiment may also have been due to much lower variability in the backwards digit span in this experiment compared to the online experiment (see Table 4). Third, it is possible that the backwards digit span was not a complex enough measure of working memory capacity to successfully capture the processes needed for perceiving tone-vocoded sentences. More complex tasks, such as the reading span test (Daneman and Carpenter, 1980) and size comparison tasks (Sörqvist et al., 2012), may capture these processes better and thus have more predictive value than digit span tasks (Daneman and Hannon, 2001; Rönnberg et al., 2019). The sentence final word identification and recall (SWIR) test, a complex auditory-based task, may also be a better method of indexing working memory resource capacity in the context of LE (Ng et al., 2013, 2015; Bönitz et al., 2021).

### What impact does experimental setting have on performance and indices of LE and tiredness in a speech recognition task?

Most effects of listening demand on performance and indices of LE observed in the laboratory experiment were not replicated in the online experiment. We used 3- and 4-band vocoder settings in the online setting, whereas the laboratory study used 2- and 3-band vocoder settings (see Methods). After piloting, the vocoder settings were modified for the online experiment so that the correct response rates were more closely aligned with the laboratory experiment. Differences in performance between laboratory and online studies using identical stimuli is not unusual. Other studies comparing performance in online and laboratory settings using identical stimuli demonstrated differences. For example, Bianco et al. (2021) found poorer performance on a speech-in-noise task performed by an online cohort compared to a group of age-matched participants who completed the same speech task in a laboratory setting. We however pursued a different strategy and sought to align performance levels as closely as possible. We chose this strategy because the *targeted* correct response rate in theory should influence motivation and listening effort, according to Motivational Intensity Theory (Brehm and Self, 1989), and we wanted motivation to be as comparable as possible between the two experimental settings. This however meant that the number of bands differed between the two experimental settings. Less control over the participants’ listening environments, listener engagement and the equipment used for listening tasks (e.g., the quality of headphones) (Clifford and Jerit, 2014; Chandler and Paolacci, 2017; Bianco et al., 2021; Zhao et al., 2022) may be part of a trade off for rapid data collection and easy access to a wide range of participants in online studies. If highly controlled stimulus presentation is needed for a particular listening tasks, online testing may not be the optimal choice of data collection.

Three additional caveats to our findings are that (a) independent groups of participants carried out each experiment, although both groups were young (18–36 years old) listeners with normal hearing (assessed using the HearWHO online and with PTA in the laboratory) (b) in the laboratory setting the presentation level of the stimuli was calibrated but in the online setting, participants were asked to play the stimuli at a comfortable listening level and (c) phonological/semantic foils used in the experiments were generated by an online rhyming dictionary, which does not clearly state the criteria on which the similarity ratings they provide are based. Further research is recommended to gain greater clarity on the feasibility of adapting laboratory-developed listening tasks to online settings, ideally involving within-groups comparisons using the same listening task.

For self-rated work, there was a larger effect of motivation in the online than in the laboratory study. This may be due to the different recruitment strategies that were used for online versus laboratory experiments. In the online study, participants may have been additionally motivated by the potential loss of financial payment and exclusion from other research studies: Prolific.co makes participants aware that they withhold payments for participation if the researcher deems their data to be of low quality (e.g., if they fail basic attention checks or provide inconsistent responses), whereas the participant information sheet for the laboratory experiment informed participants that they would receive an honorarium payment with no mention of data quality. Differences in the amount of payment offered to participants in the online experiment versus the laboratory experiment may also have influenced motivation: participants in the laboratory received a £15 honorarium while participants performing the online experiment received a £15 honorarium plus an additional £6.50 per hour (with a task duration of approximately 40 min). Additionally, Prolific.co informs participants that submitting low-quality data may preclude them from further participation opportunities (other online platforms may not do this). These factors may have increased the effectiveness of the evaluative threat manipulation in the online experiment, although effects of motivation were found for self-rated work only. Evaluative threat may need to be combined with other motivational factors, e.g., evaluative feedback (see Zekveld et al., 2019) to measure larger and broader effects of motivation on LE outcomes. Other motivational factors may also be more effective in an online setting, e.g., Bianco et al. (2021) suggest that financial reward may be particularly motivating in research conducted online as they found that offering a performance-related financial bonus improved performance in an online setting.

## Conclusion

Interactive effects of motivation and listening demands on LE, as predicted by the Framework for Understanding Effortful Listening, Motivational Intensity Theory and Cognitive Energetics Theory were not found in the two experiments of the present study. Motivation was operationalised as evaluative threat but this manipulation was not optimal for the listening task. Evaluative threat may need to be combined with other factors, such as financial reward, to effectively manipulate listeners’ motivation level. A more complex assessment of working memory than the backwards digit span may be needed to observe broader effects of resource availability across outcomes. Most effects of listening demands that were found in the laboratory setting were not replicated online. This is likely due to differences in task difficulty between the two experiments, but a lack of experimenter control over stimulus presentation may also have contributed. Future research is needed to directly compare results from online and laboratory settings, ideally using a within-participants design and using identical listening demands, to understand how these contextual factors influence the effects of listening demands and motivation on listening effort.

## Data availability statement

The raw data supporting the conclusions of this article will be made available by the authors, without undue reservation.

## Ethics statement

The studies involving humans were approved by University of Manchester Ethics Committee. The studies were conducted in accordance with the local legislation and institutional requirements. The participants provided their written informed consent to participate in this study.

## Author contributions

All authors listed have made a substantial, direct, and intellectual contribution to the work and approved it for publication.

## Footnotes

^1^https://osf.io/3vbtx

^2^https://osf.io/gcvkd
